# EBV-positive intravascular large B-cell lymphoma of the liver: a case report and literature review

**DOI:** 10.1186/s13000-020-00989-x

**Published:** 2020-06-08

**Authors:** Qingjiao Li, Jinghe Li, Keda Yang, Ying Peng, Yao Xiang, Shuyuan Sun, Jian Zeng, Xin Zhang, Junpu Wang

**Affiliations:** 1 Department of Pathology, Xiangya Hospital, Central South University, 87 Xiangya Road, Kaifu District, Changsha City, 410008 Hunan Province China; 2 Department of Pathology, School of Basic Medicine, Central South University, Changsha City, Hunan Province China

**Keywords:** Intravascular large B-cell lymphoma, IVLBCL, Liver, EBV, Case report

## Abstract

**Background:**

Intravascular large B-cell lymphoma (IVLBCL) is an extremely rare subtype of diffuse large B-cell lymphoma that most commonly involves the central nervous system, skin, and bone marrow. To our knowledge, Epstein-Barr virus (EBV)-positive IVLBCL in the liver has never been reported in the literature.

**Case presentation:**

We report a case of a 65-year-old Chinese man with complaint of fever for 18 days. No obvious abnormality was found by physical examination. Laboratory findings were notable for anemia, thrombocytopenia, and elevated level of serum lactate dehydrogenase. Bone marrow on smear, biopsy, and flow cytometry revealed no lymphoma. Imaging studies showed a slightly lower density lesion in the liver with high fluorodeoxyglucose uptake and hepatosplenomegaly. Percutaneous liver biopsy revealed clustering of large atypical lymphocytes within the hepatic sinusoids. Immunohistochemically, these lymphoma cells were positive for CD20, PAX-5, MUM-1, BCL-6 and CD5, but negative for CD3 and CD10. Besides, Epstein-Barr virus-encoded RNA was detected in tumor cells by in situ hybridization. *BCL-2*, *BCL-6* and *MYC* genes were intact tested by fluorescence in situ hybridization analysis. The patient was diagnosed as IVLBCL and died after 1 month of hospitalization without receiving immunochemotherapy.

**Conclusions:**

IVLBCL of the liver is a highly rare lymphoma with nonspecific manifestations and dismal prognosis. Full recognition of its clinicopathological features will help to better diagnose this disease.

## Background

Intravascular large B-cell lymphoma (IVLBCL) is a rare variant of extranodal diffuse large B-cell lymphoma (DLBCL) characterized by the selective growth of lymphoma cells within the lumina of small to medium-sized blood vessels, particularly in capillaries [1]. The clinical manifestations of IVLBCL are various and may vary based on geographic origin of the patients. Two major patterns of IVLBCL have been described: a Western form which displays a relatively high frequency of central nervous system (CNS) and skin involvement, and an Asian form which predominantly shows bone marrow involvement, fever, hepatosplenomegaly, and thrombocytopenia [2–4]. However, IVLBCL of the liver is distinctly uncommon and has been rarely described.

Here we report the first case of hepatic IVLBCL with EBV-positivity diagnosed by percutaneous liver biopsy. Based on literature review, we described and summarized the clinicopathological features of IVLBCL of the liver, and discussed the connection of EBV and disease prognosis.

## Case presentation

**Fig. 1:**
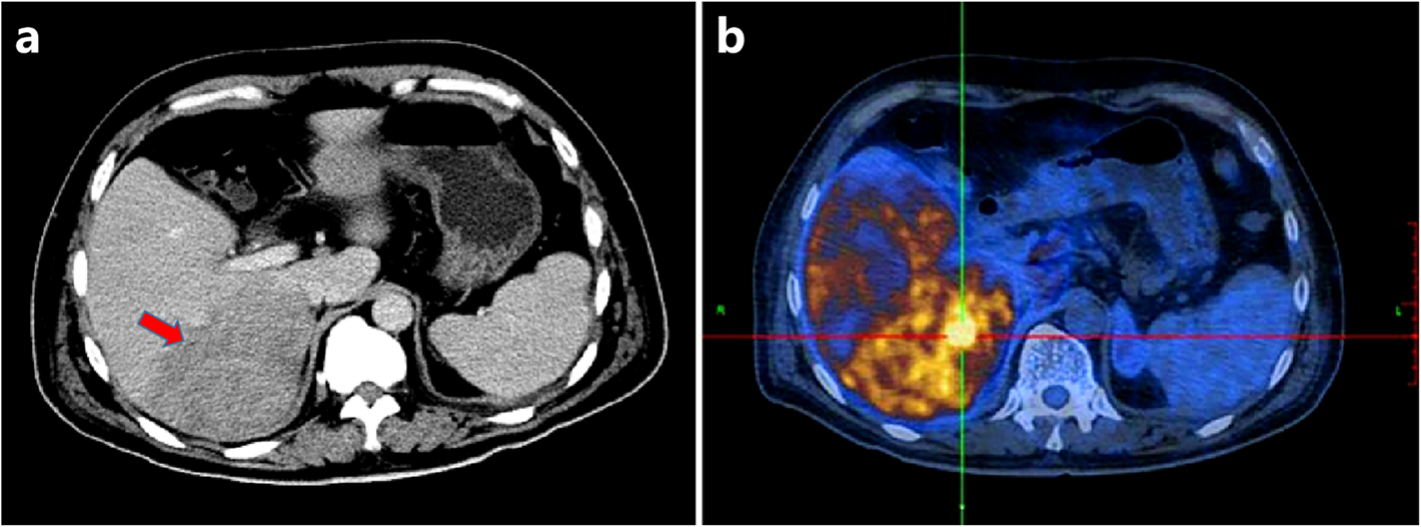
**a** Abdominal CT showed a slightly lower density lesion of 11.5 × 4.8 cm (arrow) in the right liver lobe with hepatosplenomegaly. **b** PET-CT revealed abnormal fluorodeoxyglucose uptake in the liver

A 65-year-old Chinese man with complaint of high spiking fever of unknown origin for 18 days was admitted to Xiangya Hospital, Central South University, Hunan, China. The medical history of the patient was not remarkable. No obvious abnormality was found by physical examination, including lymphadenopathy, skin lesions, or abnormal neurological signs. Abnormal laboratory findings were as follows: red blood cells, 3.0 (4.3–5.8 × 10^12^ /L); platelets, 91 (125–300 × 10^9^ /L); hemoglobin, 85 (130–175 g/L); serum albumin, 25 (40–55 g/L); serum lactate dehydrogenase (LDH), 2300 (109–245 U/L); C-reactive protein (CRP), 139 (0–8 mg/L); ferritin, > 2000 (10–240 mg/L); alanine aminotransferase (ALT), 109 (9–50 U/L); aspartate aminotransferase (AST), 160 (15–40 U/L). All numbers in parentheses mentioned above indicated the reference interval. The patient underwent an exhaustive infectious disease work-up including viral hepatitis, cytomegalovirus, herpes simplex virus, and human immunodeficiency virus, and all of the results were negative. Besides, the result of DNA quantitative study of EBV was undetectable. Bone marrow on smear, biopsy, and flow cytometry revealed no evidence of lymphomatous cells. Abdominal computed tomography (CT) showed a slightly lower density lesion of 11.5 × 4.8 cm in the right liver lobe (Fig. 1a) with hepatosplenomegaly. Positron emission tomography-computed tomography (PET-CT) revealed abnormal fluorodeoxyglucose (FDG) uptake in the liver without other organ involvement (Fig. 1b). Ultrasonography-guided percutaneous liver biopsy was performed under the impression of a suspected unusual malignant tumor.

**Fig. 2:**
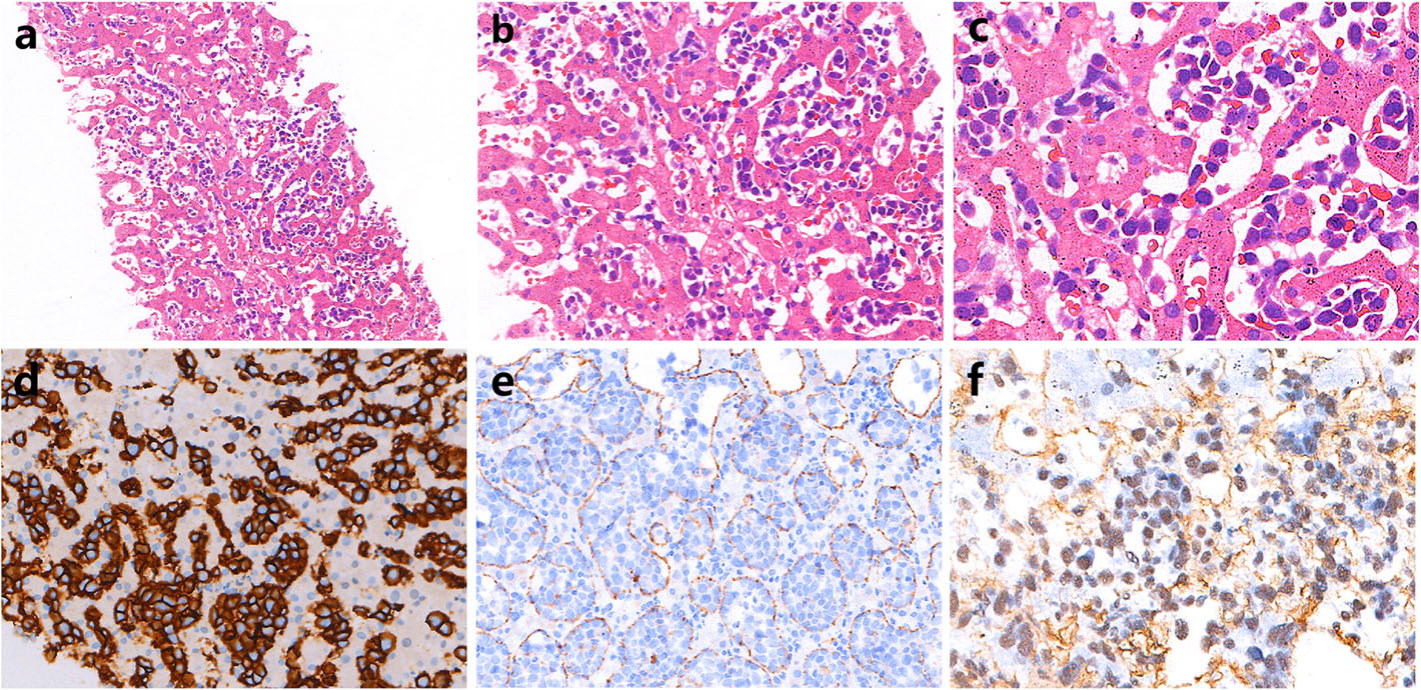
**a & b** Histopathology analysis showed the hepatic sinusoids were markedly expanded by the selective proliferation of atypical lymphocytes (**a:** magnification × 100 and **b:** magnification × 200). **c.** The neoplastic cells were large in size with round to oval-shaped nuclei and scant cytoplasm (magnification × 400). **d.** The tumor cells were positive for CD20 (magnification × 200). **e.** CD31 immunohistochemical staining highlights the intravascular growth pattern of tumor cells (magnification × 200). **f.** The neoplastic cells were positive for EBER by in situ hybridization

Histologically, the hepatic sinusoids were markedly expanded by the selective proliferation of atypical lymphocytes (Fig. 2a & b). Under higher magnification, these neoplastic lymphoid cells were large in size with round to oval-shaped nuclei, vesicular chromatin, prominent nucleoli, and scant cytoplasm (Fig. 2c). Fibrin thrombus and mitotic figures could be easily found. Immunohistochemical staining revealed the tumor cells were positive for CD20 (Fig. 2d), PAX-5, MUM-1, BCL-6 and CD5, and negative for CD3, CD10, CD56, CD38, CD138, MPO, BCL-2, HHV8, CyclinD1, and SOX11 (See Additional file 2: Figure S1). The Ki-67 proliferation index was estimated to be 80%. The intravascular growth pattern of tumor cells was confirmed by CD31 staining of the endothelial cells (Fig. 2e). The results of immunohistochemical staining were summarized in Table 1. In addition, the neoplastic cells were positive for Epstein-Barr virus-encoded small RNA (EBER) by in situ hybridization (Fig. 2f). Fluorescence in situ hybridization (FISH) tests were performed on formalin-fixed, paraffin-embedded tissues using *BCL-2*, *BCL-6* and *MYC* dual-color break-apart probes (Abbott Molecular Inc., USA), and no obvious split signals were observed with each probe.

**Table 1:** List of antibodies

Antibody	Clone	Dilution	Source	Result
CD20	L26	Ready-to-use	Maixin China	+
PAX-5	ZP007	Ready-to-use	Zhongshan China	+
MUM-1	EP190	Ready-to-use	Zhongshan China	+
BCL-6	MX042	Ready-to-use	Maixin China	+
BCL-2	bcl-2/100/D5	Ready-to-use	Zhongshan China	–
CD3	UMAB54	Ready-to-use	Zhongshan China	–
CD5	UMAB9	Ready-to-use	Zhongshan China	+
CD10	UMAB235	Ready-to-use	Zhongshan China	–
CD56	MX039	Ready-to-use	Maixin China	–
CD38	SPC32	Ready-to-use	Zhongshan China	–
CD138	EP201	Ready-to-use	Zhongshan China	–
MPO	/	Ready-to-use	Zhongshan China	–
CD31	UMAB30	Ready-to-use	Zhongshan China	–
HHV8	13B10	Ready-to-use	Zhongshan China	–
CyclinD1	EP12	Ready-to-use	Zhongshan China	–
SOX11	MRQ-58	Ready-to-use	Zhongshan China	–
Ki-67	MX0006	Ready-to-use	Maixin China	80%

Based on these findings, the patient was diagnosed as IVLBCL of the liver with EBV-positivity. The patient was treated with antibiotics initially, and then dexamethasone and supportive treatment. However, the patient’s symptoms couldn’t be obviously relieved. Because of the rapidly deteriorating general conditions, the patient died from the disease after 1 month of hospitalization without receiving immunochemotherapy.

## Discussion

IVLBCL has been described in an increasing number of reports, mostly single case report and small series. It primarily affects elderly individuals with a slight predominance in men, and can progressively involve any organ without involvement of lymphoid tissues and peripheral blood [5, 6]. Patients with IVLBCL usually present with a wide range of clinical manifestations that are generalized and nonspecific, or localized and related to the involved organ [4]. Most of the symptoms and signs might be related to organ dysfunction caused by occlusion of small vessels or capillaries. Due to the absence of significant mass lesions or lymphadenopathy, the clinical picture is further complicated and a timely diagnosis is very challenging.

Previously, one-half of IVLBCL cases were made by post-mortem [2]. With the improvement of awareness of this entity, most patients were diagnosed by bone marrow biopsy and skin biopsy from positive skin lesions or random skin biopsy [4, 7]. In our case, bone marrow biopsy revealed no evidence of lymphoma infiltration, and random skin biopsy was not performed because of a low index of suspicion of IVLBCL. Therefore, a biopsy from an affected organ is necessary. Significantly, PET-CT is a powerful tool for the early diagnosis of IVLBCL by identifying indicated sites for biopsy because these patients usually show high FDG uptake in involved organs [8, 9]. With the review of literature, liver biopsies can be performed using percutaneous, transjugular, or laparoscopic approaches, and each method has advantages and disadvantages [10]. Because of the absence of severe coagulopathy, ultrasonography-guided percutaneous liver biopsy was performed in our patient. Up to present, only 12 cases of IVLBCL diagnosed by liver biopsies have been described in the literature [10–21]. The clinical and immunohistochemical features of the reported cases and our case are summarized in Additional file 1: Table S2.

The patients with hepatic involvement of IVLBCL were predominantly men (11/13, 84.6%). The age of the patients ranged from 42 to 79 years, with a mean age of 61 years. With the exception of 2 cases from the West [14, 18], the remaining 11 cases were reported from Asia. Due to the limited number of reported cases, whether IVLBCL of liver is more prevalent among Asian populations is unclear. Lymphoma infiltration of bone marrow was detected in 4/13 patients (30.8%). The common clinical presentations were fever (7/12, 58.3%), anemia (7/12, 58.3%), thrombocytopenia (6/12, 50.0%), hepatomegaly and/or splenomegaly (8/12, 66.7%), and high levels of serum LDH (9/12, 75.0%). The ferritin and liver enzymes levels were elevated in 6 of 12 cases tested (50.0%). The clinical manifestations were variable and nonspecific, but indicative for suspected IVLBCL of the liver. The definitive diagnosis was mainly based on its typical morphologic and immunohistochemical features.

The histopathological findings presented in our case were consistent with those described in previous reports. IVLBCL of the liver was characterized by the infiltration of large atypical lymphocytes cells in the hepatic sinusoids. The neoplastic cells are typically positive for B-cell markers as CD20 and PAX-5, and a subset of cases may co-express the T-cell marker CD5 [22]. According to the algorithm of Hans et al. [23], our case was classified as the non-germinal center B-cell-like subtype (positive for BCL-6 and MUM-1, negative for CD10). Notably, EBER-positive nuclear signals were detected by in situ hybridization in our case. Although EBV-positive diffuse large B-cell lymphoma was listed as a distinct subtype of DLBCL in the revised World Health Organization (WHO) classification [1], no study focusing on EBV expression in IVLBCL has been reported. In addition, data for EBV-expression was not available in 11/12 previously reported cases, and only a case showed EBV-negativity detected by in situ hybridization [12]. The EBV-positivity rate might be higher than actual, as negative cases were less likely to be reported. It has been well established that latent EBV infection is causative of B-cell lymphoma [24]. Our case implied that EBV infection might be an important risk factor for poor prognosis of IVLBCL, like as the DLBCL. In the previously reported cases of hepatic IVLBCL, molecular characteristics of the tumor cells were not mentioned and need further investigation.

IVLBCL usually shows an aggressive clinical course with a poor prognosis. Specifically, a combination of cyclophosphamide, doxorubicin, vincristine, and prednisone with the recombinant anti-CD20 antibody rituximab (R-CHOP) is the most commonly used regimen to improve the outcome of IVLBCL [25]. Unfortunately, our patient died before treatment could be administered because of rapid disease progression.

## Conclusions

In summary, we described a rare case of IVLBCL in the liver diagnosed too late to treat the patient because of a low index of suspicion. We want to emphasize that nonspecific clinical manifestations as fever, anemia, and thrombocytopenia with markedly elevated level of serum LDH should raise the suspicion for IVLBCL and it is crucial to get histology promptly. The difficulties and delays in diagnosis often result in the poor prognosis which makes it urgent need to better understand this lymphoma. Further studies will be required to clarify the mechanisms underlying the involvement of the liver in patients with IVLBCL.

## Supplementary information

**Additional file 1: Table S2.** Clinicopathological features of previously reported and present cases of IVLBCL diagnosed by liver biopsy.

**Additional file 2: Figure S1.** The tumor cells were negative for HHV8 (**a**), CyclinD1 (**b**) and SOX11 (**c**) (**a-c:** magnification × 200). **d.** Positive control for EBER by in situ hybridization (magnification × 400).

## Data Availability

All data generated or analyzed during this case are included within the article.

## Abbreviations

IVLBCL: Intravascular large B-cell lymphoma
EBV: Epstein-Barr virus
PAX-5: Paired box protein 5
MUM-1: Multiple myeloma oncogene 1
MPO: Myeloperoxidase
DLBCL: Diffuse large B-cell lymphoma
CNS: Central nervous system
LDH: Lactate dehydrogenase
CRP: C-reactive protein
ALT: Alanine aminotransferase
AST: Aspartate aminotransferase
PET-CT: Positron emission tomography-computed tomography
FDG: Fluorodeoxyglucose
EBER: Epstein-Barr virus-encoded small RNA
FISH: Fluorescence in situ hybridization
WHO: World Health Organization
R-CHOP: Rituximab plus cyclophosphamide, doxorubicin, vincristine, and prednisone

## References

[1] Sukswai N, Lyapichev K, Khoury JD, Medeiros LJ (2020). Diffuse large B-cell lymphoma variants: an update. Pathology.

[2] Ponzoni M, Ferreri AJ, Campo E, Facchetti F, Mazzucchelli L, Yoshino T (2007). Definition, diagnosis, and management of intravascular large B-cell lymphoma: proposals and perspectives from an international consensus meeting. J Clin Oncol.

[3] Masaki Y, Dong L, Nakajima A, Iwao H, Miki M, Kurose N (2009). Intravascular large B cell lymphoma: proposed of the strategy for early diagnosis and treatment of patients with rapid deteriorating condition. Int J Hematol.

[4] Matsue K, Abe Y, Narita K, Kobayashi H, Kitadate A, Takeuchi M (2019). Diagnosis of intravascular large B cell lymphoma: novel insights into clinicopathological features from 42 patients at a single institution over 20 years. Br J Haematol.

[5] Murase T, Yamaguchi M, Suzuki R, Okamoto M, Sato Y, Tamaru J (2007). Intravascular large B-cell lymphoma (IVLBCL): a clinicopathologic study of 96 cases with special reference to the immunophenotypic heterogeneity of CD5. Blood.

[6] Ferreri AJ, Campo E, Seymour JF, Willemze R, Ilariucci F, Ambrosetti A (2004). Intravascular lymphoma: clinical presentation, natural history, management and prognostic factors in a series of 38 cases, with special emphasis on the ‘cutaneous variant’. Br J Haematol.

[7] Matsue K, Asada N, Odawara J, Aoki T, Kimura S, Iwama K (2011). Random skin biopsy and bone marrow biopsy for diagnosis of intravascular large B cell lymphoma. Ann Hematol.

[8] Miura Y, Tsudo M (2010). Fluorodeoxyglucose-PET/CT for diagnosis of intravascular large B-cell lymphoma. Mayo Clin Proc.

[9] Wagner T, Brechemier D, Dugert E, Le Guellec S, Julian A, Hitzel A (2012). Diffuse pulmonary uptake on FDG-PET with normal CT diagnosed as intravascular large B-cell lymphoma: a case report and a discussion of the causes of diffuse FDG uptake in the lungs. Cancer Imaging.

[10] Kim MJ, Park HS, Yhim HY (2017). Intravascular large b-cell lymphoma diagnosed via transjugular liver biopsy in a patient with liver dysfunction and thrombocytopenia: a case report. Medicine (Baltimore).

[11] Bae J, Lim HK, Park HY (2015). Imaging findings for intravascular large B-cell lymphoma of the liver. Clin Mol Hepatol.

[12] Sekiguchi N, Joshita S, Yoshida T, Kurozumi M, Sano K, Nakagawa M (2013). Liver dysfunction and thrombocytopenia diagnosed as intravascular large B-cell lymphoma using a timely and accurate transjugular liver biopsy. Intern Med.

[13] Makino K, Nakata J, Kawachi S, Hayashi T, Nakajima A, Yokoyama M (2013). Treatment strategy for reducing the risk of rituximab-induced cytokine release syndrome in patients with intravascular large B-cell lymphoma: a case report and review of the literature. J Med Case Rep.

[14] Rashidi A, Roullet MR (2012). Intravascular large B-cell lymphoma with leukemic component. Blood.

[15] Fung KM, Chakrabarty JH, Kern WF, Magharyous H, Gehrs BC, Li S (2012). Intravascular large B-cell lymphoma with hemophagocytic syndrome (Asian variant) in a Caucasian patient. Int J Clin Exp Pathol.

[16] Aoki Y, Takamiya M, Satoh T, Fujita S, Kato H, Maeno Y (2011). A fatal case of hemoperitoneum after ultrasound-guided liver biopsy in a patient with intravascular large B-cell lymphoma. Leg Med (Tokyo).

[17] Hsieh MS, Yeh YC, Chou YH, Lin CW (2010). Intravascular large B cell lymphoma in Taiwan: an Asian variant of non-germinal-center origin. J Formos Med Assoc.

[18] Roshal M, Till BG, Fromm JR, Cherian S (2008). Intravascular large B cell lymphoma presenting in a liver explant. J Clin Pathol.

[19] Shiraki K, Sugimoto K, Deguchi M, Ito N, Masuda C, Takei Y (2007). Hepatic intravascular large B cell lymphoma. Intern Med.

[20] Tokura T, Murase T, Toriyama T, Totani Y, Negita M, Akaza K (2003). Asian variant of CD5+ intravascular large B-cell lymphoma with splenic infarction. Intern Med.

[21] Abe H, Kamimura K, Mamizu M, Shibazaki Y, Ishiguro T, Katada S (2014). Early diagnosis of hepatic intravascular lymphoma: a case report and literature review. Intern Med.

[22] Yamamoto K, Yakushijin K, Okamura A, Hayashi Y, Matsuoka H, Minami H (2013). Gain of 11q by an additional ring chromosome 11 and trisomy 18 in CD5-positive intravascular large B-cell lymphoma. J Clin Exp Hematop.

[23] Hans CP, Weisenburger DD, Greiner TC, Gascoyne RD, Delabie J, Ott G (2004). Confirmation of the molecular classification of diffuse large B-cell lymphoma by immunohistochemistry using a tissue microarray. Blood.

[24] Zhang Y, Bi L, Qiu Y, Zhao T, Cao M, Ding J (2018). Primary pulmonary intravascular large B-cell lymphoma: a report of three cases and literature review. Oncol Lett.

[25] Shimada K, Kosugi H, Narimatsu H, Shimada S, Suzuki T, Ito M (2008). Sustained remission after rituximab-containing chemotherapy for intravascular large B-cell lymphoma. J Clin Exp Hematop.

