# Supplementary figures and images for: EBV-positive intravascular large B-cell lymphoma of the liver: a case report and literature review
Source: Diagn Pathol. 2020 Jun 8;15:72. doi: 10.1186/s13000-020-00989-x (PMC7282072; doi:10.1186/s13000-020-00989-x)

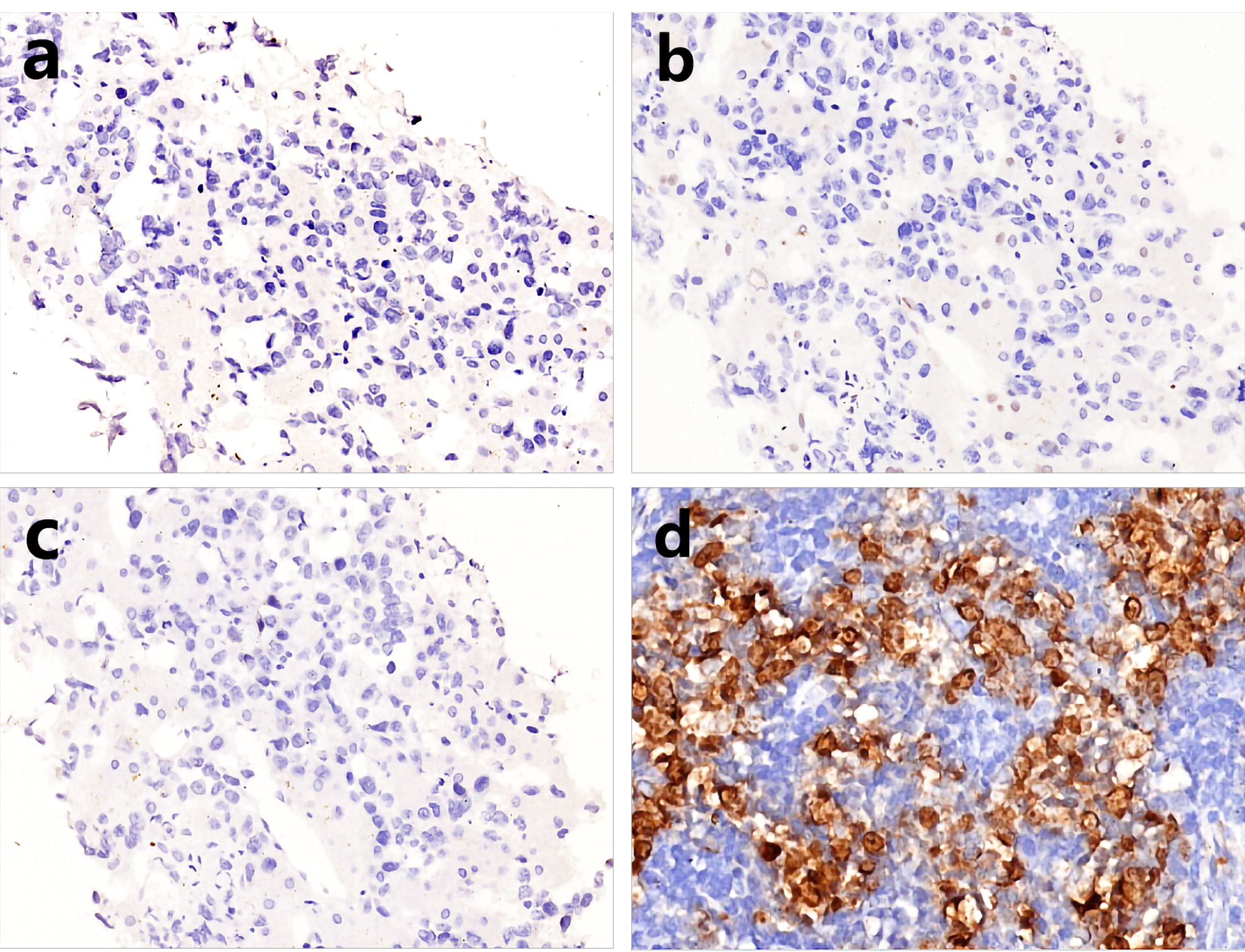

Supplement: Supplementary file 2 — Additional file 2: Figure S1. The tumor cells were negative for HHV8 (a), CyclinD1 (b) and SOX11 (c) (a-c: magnification × 200). d. Positive control for EBER by in situ hybridization (magnification × 400). [file 13000_2020_989_MOESM2_ESM.tif]
